# Early Complication Differences Between Laparoscopic and Open Abdominoperineal Resection

**DOI:** 10.7759/cureus.94533

**Published:** 2025-10-14

**Authors:** Sarkhail A Sayar, Rehan Ahmed, Syed Shafqatullah, Muhammad Asad, Muhammad Osama Iqbal, Resham Ali, Raja Jawad, Mukkaram Khan, Amna Fareed, Uroosa Shadani

**Affiliations:** 1 General and Colorectal Surgery, Jinnah Postgraduate Medical Centre, Karachi, PAK; 2 General Surgery, IIMCT-Pakistan Railway Hospital, Rawalpindi, PAK; 3 Surgery, Riphah International University, Rawalpindi, PAK

**Keywords:** laparoscopic abdominoperineal resection, minimally invasive surgery, open abdominoperineal resection, postoperative complications, rectal cancer

## Abstract

Background

Abdominoperineal resection (APR) is a common surgical approach for low rectal cancer (RC). With advancements in minimally invasive techniques, laparoscopic APR (LAPR) has gained popularity. However, comparative data on early postoperative complications between laparoscopic and open APR (OAPR) remain limited at Jinnah Postgraduate Medical Centre (JPMC), Karachi.

Objective

The primary endpoint of the study was to evaluate any 30-day postoperative complications. Secondary endpoints included surgical site infection (SSI), pulmonary complications, intraoperative blood loss, operative time, and length of hospital stay. The study aimed to compare these outcomes between laparoscopic and OAPR in patients with low RC. It was hypothesized that patients undergoing LAPR would have fewer early postoperative complications than those undergoing OAPR.

Methodology

This prospective, comparative observational study was conducted at JPMC, Karachi, from January 2024 to January 2025. A total of 90 patients diagnosed with low rectal adenocarcinoma were enrolled and divided equally into two groups: LAPR (Group A) and OAPR (Group B). Standardized surgical techniques were employed in both groups. Patients were monitored for 30 days postoperatively for complications such as SSIs, pulmonary issues, ileus, and reoperations. Statistical analysis was performed using IBM SPSS Statistics for Windows, Version 26.0 (Released 2018; IBM Corp., Armonk, NY, USA), with significance set at p < 0.05.

Results

The laparoscopic group had a longer mean operative time than the open group (195.2 ± 23.4 vs. 158.7 ± 20.1 minutes, p < 0.001) but showed reduced intraoperative blood loss (120.5 ± 40.8 vs. 285.3 ± 60.2 mL, p < 0.001), shorter hospital stay (6.2 ± 1.4 vs. 9.3 ± 2.1 days, p < 0.001), and fewer overall complications (20% vs. 51.1%, relative risk 0.39, p = 0.002). SSIs (8.9% vs. 24.4%, RR 0.37, p = 0.047) and pulmonary complications (2.2% vs. 13.3%, RR 0.17, p = 0.048) were also significantly lower in the laparoscopic group.

Conclusions

In this single-center prospective cohort, LAPR was associated with fewer early postoperative complications, lower blood loss, and shorter hospital stays than OAPR, despite longer operative time. These findings suggest that LAPR may be a reasonable option in appropriately selected patients, but confirmation in multicenter randomized studies with long-term oncologic outcomes is warranted.

## Introduction

Abdominoperineal resection (APR) is a surgical procedure performed on patients with anorectal or distal rectal cancer (RC) when an anterior resection is not feasible to preserve the anal sphincter [1]. Originally described by Ernest Miles in 1908 [2], APR has evolved with the advent of laparoscopic techniques, which are increasingly used for RC surgery [3,4].

Laparoscopic techniques are generally associated with advantages over open surgery, such as reduced postoperative pain, shorter ileus duration, decreased hospital stay, faster recovery, and earlier return to daily activities [5-7]. However, much of the supporting evidence comes from broader colorectal or minimally invasive surgery literature rather than specifically from APR, and findings should therefore be interpreted with caution in the context of RC surgery.

Colorectal cancer is the third most commonly diagnosed cancer worldwide and the second leading cause of cancer-related mortality [8]. Despite advancements in multimodal treatment strategies, aggressive surgical intervention remains the cornerstone for locally advanced RC. Innovations such as total mesorectal excision (TME) and neoadjuvant chemoradiotherapy (NCRT) have significantly improved treatment outcomes in recent years [9].

Surgical options under TME include APR, low anterior resection (LAR), and intersphincteric resection (ISR) [10-13]. Although the use of APR has declined with the increasing adoption of LAR and ISR, it remains a necessary surgical option for approximately 30% of patients with low-lying RC [14].

Recent studies have shown that laparoscopic APR (LAPR) is comparable to open APR (OAPR) in terms of oncologic outcomes. In a large randomized trial including 1,076 eligible patients (534 laparoscopic and 542 open), median follow-up was 53 months. The combined three-year disease-free survival (DFS) for all stages was 74.2% in the laparoscopic group versus 76.2% in the open-surgery group (p = 0.70; HR 0.92, 95% CI 0.74-1.15). Similarly, three-year overall survival (OS) was 81.8% for laparoscopic versus 84.2% for open surgery (p = 0.45; HR 0.95, 95% CI 0.74-1.22). Resection margins, number of lymph nodes retrieved, morbidity, and mortality were also similar between groups [15]. Moreover, LAPR may overcome some limitations of OAPR, such as large abdominal incisions and a higher risk of surgical site infections (SSIs). A recent meta-analysis confirmed the short-term benefits of LAPR, including reduced intraoperative blood loss, shorter hospital stays, and fewer postoperative complications, particularly wound infections [16].

However, earlier studies that included ISR and LAR were limited by small sample sizes and lacked robust long-term oncological outcomes [14,17]. The American College of Surgeons Oncology Group Z6051 randomized trial failed to confirm the non-inferiority of laparoscopy compared with open surgery for RC in terms of pathological outcomes, with APR performed in a subset of patients. Similarly, the ALaCaRT trial, which included patients with T1-T3 rectal tumors, also did not establish the non-inferiority of laparoscopic surgery for successful resection [18,19]. The advantages of LAPR and OAPR for RC patients, therefore, remain disputed. Although both trials demonstrated generally high-quality surgery, their findings do not provide sufficient evidence to support the routine use of laparoscopy for RC without longer-term data on recurrence and survival.

This study aimed to evaluate and compare the early postoperative outcomes of laparoscopic versus OAPR in patients with low RC. By focusing on short-term complications within 30 days after surgery, it sought to determine whether the minimally invasive approach is associated with improved perioperative recovery compared with the conventional open technique.

## Materials and methods

This prospective, comparative observational study was conducted in the Department of General Surgery at Jinnah Postgraduate Medical Centre, Karachi, from January 2024 to January 2025, using consecutive sampling to enroll patients. The objective of the study was to evaluate and compare the early postoperative complications in patients undergoing laparoscopic versus OAPR for low RC. The primary endpoint was the overall incidence of early postoperative complications occurring within 30 days of surgery. Secondary endpoints included the occurrence of specific complications such as SSI, wound dehiscence, postoperative ileus, UTI, pulmonary complications, venous thromboembolism (VTE), bleeding requiring intervention, and reoperation within 30 days.

Prior to initiation, ethical approval was secured from the College of Physicians and Surgeons Pakistan (CPSP) Research and Ethics Committee under approval number CPSP/REU/SGR-2022-186-13857, dated November 24, 2023. Informed written consent was obtained from all participants.

The sample size was calculated using OpenEpi version 3.0, based on a confidence level of 95%, statistical power of 80%, and estimated differences in early complication rates from previous literature. The minimum required sample was 90 patients in total. These patients were equally divided into two groups: Group A underwent LAPR (n = 45), while Group B underwent OAPR (n = 45).

Participants were selected using a non-probability consecutive sampling technique. All patients aged between 18 and 75 years with biopsy-proven adenocarcinoma of the distal rectum (tumor located ≤5 cm from the anal verge) and scheduled for elective curative APR were considered eligible. Only patients classified as American Society of Anesthesiologists (ASA) physical status I-III and with resectable tumors on preoperative imaging (pelvic MRI and staging CT) were included. Patients who had completed NCRT at least six weeks before surgery were also included.

Exclusion criteria were patients undergoing emergency surgery, those with evidence of distant metastasis, previous extensive abdominal or pelvic surgery that precluded laparoscopic access, inflammatory bowel disease, synchronous malignancies, contraindications to general anesthesia, or those who declined to give consent. Patients lost to follow-up within 30 days postoperatively were also excluded from the final analysis.

All surgeries were performed by consultant general surgeons with standardized training and experience of at least 10 years in colorectal surgery. LAPR involved standard port placement (typically four to five ports), high ligation of the inferior mesenteric vessels, TME, mobilization of the sigmoid colon, and perineal dissection followed by end colostomy. The OAPR group underwent a midline laparotomy, with similar oncologic principles, including TME and perineal dissection. Perioperative care (antibiotic prophylaxis, thromboembolic prophylaxis, postoperative pain management, and enhanced recovery protocols) was standardized across both groups.

For patients initially scheduled for LAPR, conversion to open surgery was undertaken at the discretion of the operating surgeon in cases of uncontrolled bleeding, poor visualization, difficult pelvic anatomy, dense adhesions, or inability to safely progress with laparoscopic dissection. All conversions were documented, and patients were analyzed in the laparoscopic intention-to-treat group.

Postoperative monitoring included daily clinical assessments, laboratory investigations, and imaging when indicated. Patients were followed for 30 days postoperatively to monitor early complications, which were defined as any complication occurring within this time frame. Complications recorded included SSIs (superficial, deep, or organ-space), UTIs, wound dehiscence, postoperative ileus (defined as no bowel movement beyond 72 hours), pulmonary complications (such as pneumonia or atelectasis), VTE, bleeding requiring intervention, and the need for reoperation. The severity of complications was classified according to the Clavien-Dindo grading system. Outcomes were assessed from clinical documentation by the treating surgical team; blinding of outcome assessors was not performed.

All data were entered and analyzed using IBM SPSS Statistics for Windows, Version 26.0 (Released 2018; IBM Corp., Armonk, NY, USA). Quantitative variables such as age, duration of surgery, and hospital stay were reported as mean ± SD. Categorical variables such as gender, ASA grade, tumor location, and type and frequency of complications were expressed as frequencies and percentages. The independent sample t-test was applied to compare continuous variables between the two groups, while categorical variables were analyzed using the chi-square test or Fisher’s exact test, where appropriate. A p-value of less than 0.05 was considered statistically significant.

## Results

The demographic and baseline clinical characteristics of the two groups are shown in Table 1. No statistically significant differences were observed in baseline characteristics between the two groups, indicating adequate group comparability (p > 0.05 for all).

**Table 1:** Baseline demographic and clinical characteristics of patients (n = 90) Values are expressed as mean ± SD or n (%). The independent t-test was used for continuous variables, and the chi-square test was used for categorical variables. Test statistics (t-values and χ² values) are shown in parentheses. LAPR, laparoscopic abdominoperineal resection; OAPR, open abdominoperineal resection

Variable	LAPR (n = 45)	OAPR (n = 45)	p-Value
Age (years), mean ± SD	56.7 ± 8.9	58.1 ± 9.4	0.415 (t = -0.73)
Gender (male/female), n (%)	28 (62.2%)/17 (37.8%)	30 (66.7%)/15 (33.3%)	0.653 (χ² = 0.05)
BMI (kg/m²), mean ± SD	25.4 ± 3.1	25.8 ± 3.5	0.544 (t = -0.57)
ASA Class I/II/III, n (%)	12 (26.7%)/23 (51.1%)/10 (22.2%)	10 (22.2%)/25 (55.6%)/10 (22.2%)	0.889 (χ² = 0.27)
Tumor distance from anal verge (cm), mean ± SD	3.2 ± 1.0	3.4 ± 1.1	0.412 (t = -0.90)
Completion of neoadjuvant therapy (Yes), n (%)	31 (68.9%)	29 (64.4%)	0.655 (χ² = 0.05)

Table 2 highlights intraoperative and perioperative outcomes. The LAPR group had significantly longer operative time (195.2 ± 23.4 vs. 158.7 ± 20.1 minutes, p < 0.001), but showed markedly reduced intraoperative blood loss (120.5 ± 40.8 mL vs. 285.3 ± 60.2 mL, p < 0.001) and shorter hospital stay (6.2 ± 1.4 vs. 9.3 ± 2.1 days, p < 0.001). Conversion to open surgery occurred in two patients (4.4%) due to dense adhesions.

**Table 2:** Intraoperative and perioperative parameters ^*^ p < 0.05 is considered statistically significant. Values are expressed as mean ± SD or n (%). The independent t-test was used for continuous variables, and the chi-square test was used for categorical variables. Test statistics (t-values and χ² values) are shown in parentheses. LAPR, laparoscopic abdominoperineal resection; OAPR, open abdominoperineal resection

Parameter	LAPR (n = 45)	OAPR (n = 45)	p-Value
Duration of surgery (minutes), mean ± SD	195.2 ± 23.4	158.7 ± 20.1	<0.001^*^ (t = 7.94)
Intraoperative blood loss (mL), mean ± SD	120.5 ± 40.8	285.3 ± 60.2	<0.001^*^ (t = -15.20)
Length of hospital stay (days), mean ± SD	6.2 ± 1.4	9.3 ± 2.1	<0.001^* ^(t = -8.24)
Conversion to open surgery, n (%)	2 (4.4%)	-	- (χ² = 0.51)

Early postoperative complications are shown in Table 3. The laparoscopic group experienced a significantly lower rate of overall complications (20%) compared to the open group (51.1%), with a statistically significant p-value (p = 0.002). SSIs were significantly less frequent in the laparoscopic group (8.9% vs. 24.4%, p = 0.047), as were pulmonary complications (2.2% vs. 13.3%, p = 0.048). Wound dehiscence (2.2% vs. 11.1%, p = 0.092) and postoperative ileus (6.7% vs. 20%, p = 0.058) showed a trend toward higher rates in the open group, although these differences did not reach statistical significance. The laparoscopic-to-open conversion rate was 4.4%, which is comparable to rates reported in contemporary series (approximately 3-10%) of LAPR.

**Table 3:** Early postoperative complications within 30 days ^*^ p < 0.05 is considered statistically significant. LAPR, laparoscopic abdominoperineal resection; OAPR, open abdominoperineal resection; SSI, surgical site infection; VTE, venous thromboembolism

Complication	LAPR (n = 45)	OAPR (n = 45)	RR (95% CI)	p-Value
Any complication (overall)	9 (20%)	23 (51.1%)	0.39 (0.20-0.73)	0.002^*^
SSI	4 (8.9%)	11 (24.4%)	0.36 (0.13-0.97)	0.047^*^
Wound dehiscence	1 (2.2%)	5 (11.1%)	0.20 (0.02-1.63)	0.092
Postoperative ileus	3 (6.7%)	9 (20%)	0.33 (0.10-1.09)	0.058
UTI	2 (4.4%)	5 (11.1%)	0.40 (0.08-1.90)	0.231
Pulmonary complications	1 (2.2%)	6 (13.3%)	0.17 (0.02-1.30)	0.048^*^
VTE	1 (2.2%)	3 (6.7%)	0.33 (0.04-2.92)	0.308
Reoperation within 30 days	0 (0%)	2 (4.4%)	0.39 (0.20-0.73)	0.153

The severity of complications graded by the Clavien-Dindo classification is shown in Table 4. The laparoscopic group had fewer severe complications (Grade III or higher) and a higher number of patients without any complications (80% vs. 48.9%, p = 0.002). Although individual grades did not show significant differences, trends consistently favored LAPR.

**Table 4:** Clavien-Dindo classification of complications ^*^ p < 0.05 is considered statistically significant. Values are expressed as mean ± SD or n (%). The independent t-test was used for continuous variables, and the chi-square test was used for categorical variables. Test statistics (t-values and χ² values) are shown in parentheses.

Clavien-Dindo Grade, n (%)	LAPR (n = 45)	OAPR (n = 45)	p-Value
Grade I	3 (6.7%)	7 (15.6%)	0.181
Grade II	5 (11.1%)	10 (22.2%)	0.150
Grade IIIa	1 (2.2%)	4 (8.9%)	0.170
Grade IIIb or higher	0 (0%)	2 (4.4%)	0.153
No complication	36 (80%)	22 (48.9%)	0.002^*^ (χ² = 8.20)

## Discussion

This comparative study evaluated early postoperative outcomes between LAPR and OAPR in patients undergoing curative surgery for low rectal carcinoma. Our findings suggested significant differences in perioperative outcomes and complication rates between the two groups, with the laparoscopic approach demonstrating several advantages, despite longer operative duration.

The mean operative duration was significantly longer in the laparoscopic group (195.2 ± 23.4 minutes) compared to the open group (158.7 ± 20.1 minutes), consistent with prior studies attributing prolonged laparoscopic times to the learning curve and technical challenges in confined pelvic spaces [16]. However, despite this longer operative duration, the laparoscopic approach was associated with a lower risk of any complication within 30 days compared to OAPR (relative risk 0.39, 95% CI 0.20-0.75), indicating reduced short-term morbidity. This reduction may reflect improved visualization and dissection and/or unmeasured differences in surgical devices, hemostatic strategies, or surgeon technique [20,21]. Clinically, reduced blood loss may decrease transfusion requirements and associated complications.

Patients undergoing LAPR also experienced shorter postoperative hospital stays (6.2 ± 1.4 days vs. 9.3 ± 2.1 days), in line with previous randomized trials and meta-analyses [22,23]. This likely reflects decreased postoperative pain, earlier mobilization, faster return of bowel function, and the overall minimally invasive nature of the procedure.

The overall 30-day complication rate was significantly lower in the laparoscopic group (20.0% vs. 51.1%, p = 0.002) [24-26]. Notably, SSIs were less frequent in the laparoscopic group (8.9% vs. 24.4%, p = 0.047), consistent with reduced incision length and less exposure of intra-abdominal contents [27-29]. All patients underwent primary perineal wound closure according to hospital protocol. Pulmonary complications were also lower (2.2% vs. 13.3%, p = 0.048), likely reflecting improved respiratory mechanics, reduced postoperative pain, and earlier ambulation [30]. While wound dehiscence, UTIs, postoperative ileus, and VTE did not reach statistical significance, they all trended favorably toward laparoscopy, supporting its clinical benefit. Reoperation within 30 days occurred only in the open group (4.4%), further highlighting improved short-term outcomes.

According to the Clavien-Dindo grading system, the laparoscopic group had fewer severe complications (Grade III or higher) and a higher proportion of patients without complications (80% vs. 48.9%, p = 0.002) [26]. Although individual complication grades did not achieve statistical significance, the consistent downward trend reinforces the clinical safety of LAPR when performed by experienced surgeons.

Strengths and limitations

A major strength of our study lies in the prospective collection and comparison of early postoperative outcomes between two clearly defined surgical cohorts. Nonetheless, certain limitations must be acknowledged. This was a non-randomized, single-center study with a relatively small sample size, which may limit generalizability and increase susceptibility to selection or surgeon-related bias. The follow-up period was limited to 30 days, precluding assessment of long-term oncological outcomes such as DFS and OS. Therefore, while this study demonstrates the short-term safety and benefits of LAPR, it cannot provide evidence regarding long-term cancer control. Additionally, surgeon experience may have influenced intraoperative and postoperative outcomes, although trained colorectal teams performed both approaches.

Clinical implications

Our findings support the use of LAPR as a reasonable option for patients with low RC in centers with experienced surgical teams. Reductions in SSIs and pulmonary complications are likely to improve postoperative recovery, reduce healthcare costs, and enhance patient quality of life. However, further multicenter randomized studies with long-term follow-up are warranted to confirm these findings and establish oncological equivalence.

## Conclusions

LAPR was associated with a lower risk of any complication within 30 days compared with OAPR (relative risk 0.39, 95% CI 0.20-0.75) in this cohort. The laparoscopic approach was also associated with reduced blood loss and shorter hospital stays, despite longer operative times. These findings suggest potential short-term benefits of LAPR; however, as a non-randomized, single-center study, they should be interpreted cautiously. Residual confounding and limited generalizability must be acknowledged, and larger multicenter randomized trials with long-term oncological follow-up are needed to confirm these results.

## References

[1] Ray-Offor E, Horesh N, Emile SH (2024). Laparoscopic abdominoperineal resection. Colorectal & Hernia Laparoscopic Surgery.

[2] Miles WE (1971). A method of performing abdomino-perineal excision for carcinoma of the rectum and of the terminal portion of the pelvic colon (1908). CA Cancer J Clin.

[3] Jacobs M, Verdeja JC, Goldstein HS (1991). Minimally invasive colon resection (laparoscopic colectomy). Surg Laparosc Endosc.

[4] Gavrila D, Bitere O, Droc G (2021). Abdominoperineal resection for rectal cancer: open, laparoscopic or robotic approach. Chirurgia (Bucur).

[5] Gezen C, Altuntas YE, Kement M (2011). Laparoscopic abdominoperineal resections for mid or low rectal adenocarcinomas: a retrospective, comparative study. Surg Laparosc Endosc Percutan Tech.

[6] Inada R, Yamamoto S, Oshiro T, Takawa M, Fujita S, Akasu T (2014). A case-matched comparison of the short-term outcomes between laparoscopic and open abdominoperineal resection for rectal cancer. Surg Today.

[7] Ng SS, Leung KL, Lee JF, Yiu RY, Li JC, Teoh AY, Leung WW (2008). Laparoscopic-assisted versus open abdominoperineal resection for low rectal cancer: a prospective randomized trial. Ann Surg Oncol.

[8] Hossain MS, Karuniawati H, Jairoun AA (2022). Colorectal cancer: a review of carcinogenesis, global epidemiology, current challenges, risk factors, preventive and treatment strategies. Cancers (Basel).

[9] Smith HG, Nilsson PJ, Shogan BD (2024). Neoadjuvant treatment of colorectal cancer: comprehensive review. BJS Open.

[10] Farouk A, Hamdy E, Fouad A, Hamed H (2024). Oncological and functional outcomes of sphincter saving procedures versus abdominoperineal resection for low rectal cancer: a comparative prospective cohort study. Egyptian J Surg.

[11] Kaderi AS, Singh S, Sharma A, Kazi M, Desouza A, Saklani A (2025). Is it worth performing intersphincteric resection in patients having rectal adenocarcinoma with oligometastasis. Indian J Surg Oncol.

[12] Shamim M (2024). Overview of the current standards in rectal carcinoma treatment. Onkol Radioter.

[13] Zedan A (2021). Intersphincteric resection for distal rectal cancer without a defunctioning stoma. EC Gastroenterol Dig Syst.

[14] Du Q, Yang W, Zhang J (2024). Oncologic outcomes of intersphincteric resection versus abdominoperineal resection for lower rectal cancer: a systematic review and meta-analysis. Int J Surg.

[15] Buunen M, Veldkamp R, Hop WC (2009). Survival after laparoscopic surgery versus open surgery for colon cancer: long-term outcome of a randomised clinical trial. Lancet Oncol.

[16] Zhang X, Wu Q, Hu T, Gu C, Bi L, Wang Z (2018). Laparoscopic versus conventional open abdominoperineal resection for rectal cancer: an updated systematic review and meta-analysis. J Laparoendosc Adv Surg Tech A.

[17] Ursi P, Santoro A, Gemini A (2018). Comparison of outcomes following intersphincteric resection vs low anterior resection for low rectal cancer: a systematic review. G Chir.

[18] Fleshman J, Branda M, Sargent DJ (2015). Effect of laparoscopic-assisted resection vs open resection of stage II or III rectal cancer on pathologic outcomes: the ACOSOG Z6051 randomized clinical trial. JAMA.

[19] Stevenson AR, Solomon MJ, Lumley JW (2015). Effect of laparoscopic-assisted resection vs open resection on pathological outcomes in rectal cancer: the ALaCaRT randomized clinical trial. JAMA.

[20] Garcia-Henriquez N, Galante DJ, Monson JR (2020). Selection and outcomes in abdominoperineal resection. Front Oncol.

[21] Cho MS, Bae HW, Kim NK (2024). Essential knowledge and technical tips for total mesorectal excision and related procedures for rectal cancer. Ann Coloproctol.

[22] Odermatt M, Flashman K, Khan J, Parvaiz A (2016). Laparoscopic-assisted abdominoperineal resection for low rectal cancer provides a shorter length of hospital stay while not affecting the recurrence or survival: a propensity score-matched analysis. Surg Today.

[23] Yan X, Su H, Zhang S (2021). Pelvic peritoneum closure reduces postoperative complications of laparoscopic abdominoperineal resection: 6-year experience in single center. Surg Endosc.

[24] Black AJ, Karimuddin A, Raval M, Phang T, Brown CJ (2021). The impact of laparoscopic technique on the rate of perineal hernia after abdominoperineal resection of the rectum. Surg Endosc.

[25] Liu ZH, Zeng ZW, Jie HQ (2022). Transanal total mesorectal excision combined with intersphincteric resection has similar long-term oncological outcomes to laparoscopic abdominoperineal resection in low rectal cancer: a propensity score-matched cohort study. Gastroenterol Rep (Oxf).

[26] Feng Q, Tang W, Zhang Z (2022). Robotic versus laparoscopic abdominoperineal resections for low rectal cancer: a single-center randomized controlled trial. J Surg Oncol.

[27] Cataneo JL, Mathis SA, Del Valle DD, Perez-Tamayo AM, Mellgren AF, Gantt G Jr, Alkureishi LW (2023). Outcomes of perineal wound closure techniques after abdominoperineal resections in rectal cancer: an NSQIP propensity score matched study. J Plast Surg Hand Surg.

[28] Kaneko T, Funahashi K, Ushigome M, Kagami S, Goto M, Koda T, Kurihara A (2021). Incisional negative pressure wound therapy to reduce perineal wound infection after abdominoperineal resection. Int Wound J.

[29] Shen Y, Yang T, Zeng H, Meng W, Wang Z (2021). Efficacy of pelvic peritoneum closure after laparoscopic extralevator abdominoperineal excision for rectal cancer. J Gastrointest Surg.

[30] Matsuda T, Yamashita K, Hasegawa H (2021). Transperineal minimally invasive abdominoperineal resection for low rectal cancer: standardized technique and clinical outcomes. Surg Endosc.

